# A comprehensive Benchmark for fake news detection

**DOI:** 10.1007/s10844-021-00646-9

**Published:** 2022-03-21

**Authors:** Antonio Galli, Elio Masciari, Vincenzo Moscato, Giancarlo Sperlí

**Affiliations:** Department of Electrical and Information Technology (DIETI), University of Naples, Federico II via Claudio 21, 80125 Naples, Italy

**Keywords:** Fake news detection, Deep learning, Benchmarking

## Abstract

Nowadays, really huge volumes of fake news are continuously posted by malicious users with fraudulent goals thus leading to very negative social effects on individuals and society and causing continuous threats to democracy, justice, and public trust. This is particularly relevant in social media platforms (e.g., Facebook, Twitter, Snapchat), due to their intrinsic uncontrolled publishing mechanisms. This problem has significantly driven the effort of both academia and industries for developing more accurate fake news detection strategies: early detection of fake news is crucial. Unfortunately, the availability of information about news propagation is limited. In this paper, we provided a benchmark framework in order to analyze and discuss the most widely used and promising machine/deep learning techniques for fake news detection, also exploiting different features combinations w.r.t. the ones proposed in the literature. Experiments conducted on well-known and widely used real-world datasets show advantages and drawbacks in terms of accuracy and efficiency for the considered approaches, even in the case of limited content information.

## Introduction

In the last decade, with the disruptive diffusion of social media, people turned towards consuming news from journalistic websites to popular social media platforms (e.g., Facebook, Twitter, Reddit) (Matsa & Shearer, 2018; Almoqbel et al., 2019; Corradini et al., 2021; 2020). As a result, we observed the diffusion of *fake news* on the Web, whose proliferation is especially due to features and publishing mechanisms of *Online Social Networks* (OSNs). More in detail, as social media are nowadays the main medium for large-scale information sharing and communication and they can be considered the main drivers of the Big Data revolution we observed in recent years (Agrawal & et al., 2012), requiring more sophisticated techniques for their analysis (Ianni et al., 2020; Masciari, 2012). The reasons for this change in consumption behaviors are inherent to the characteristics of modern OSNs: they are more convenient and less expensive compared with traditional news media (e.g. television or newspapers); it is easier to share, comment on, and discuss the news with friends and followers. Unfortunately, due to malicious user having fraudulent goals fake news on social media are growing quickly both in volume and their potential influence thus leading to very negative social effects. Thus, fake news detection on social media poses peculiar challenges due to the inherent nature of social networks that requires both the analysis of their content (Potthast et al., 2017; Guo et al., 2019) and their social context(Shu et al., 2019). In this respect, identifying and moderating fake news is a quite challenging problem. Indeed, fighting fake news in order to stem their extremely negative effects on individuals and society is crucial in many real life scenarios. Therefore, fake news detection on social media has recently become an hot research topic both for academia and industry despite many OSN websites are already adopting several techniques to stem fake news spread.

Fake news detection dates back long time ago (Zhou et al., 2019) as journalist and scientists fought against misinformation since the beginning of information sharing by traditional media. Dealing with (modern) fake news is made even more complex since there is no single accepted definition of such a concept. One the most diffused is reported in (Allcott & Gentzkow, 2017):

“*Fake news is a news article that is intentionally and verifiably false and could mislead readers*”.

We can observe how two key features clearly emerge from such a definition: fake news includes false information that can be verified as such (authenticity) and it is created with the dishonest intention to mislead consumers (intent) (Shu et al., 2017).

The content of fake news is usually heterogeneous in terms of topics, shape and media platforms, and attempts to misrepresent truth with diverse linguistic styles while simultaneously mocking true news. Moreover, fake news is generally related to newly emerging, time-critical events, which may not have been properly verified by existing knowledge bases due to the lack of confirmed evidence or claims.

Furthermore, fake news detection on social media presents unique characteristics and challenges:

- Fake news is designed and written on purpose to deceive readers to believe false information, therefore detecting a fake news through the analysis only of the news content is a difficult and nontrivial task.
- It could be convenient to include auxiliary information, such as user social interaction on social media, to help fake news detection. However, exploiting social auxiliary information is a challenge because users social interaction with fake news produce data that is big, noisy, unstructured and incomplete.

Actually, the influential power of fake news is explained by several psychological theories. Fake news mainly targets people by exploiting their vulnerabilities. There are two major factors which make consumers naturally vulnerable to fake news (Shu et al., 2017):

- *Naive Realism*: people tend to believe that their perceptions of reality are the only accurate views, while others who disagree are regarded as uninformed or irrational;
- *Confirmation Bias*: people prefer to receive information that confirms their existing views.

People are exposed to certain news owing to the way news feed appears on their OSNs’ homepage, amplifying the psychological challenges to dispelling fake news identified above. People can trust in fake news due to different psychological factors (Shu et al., 2017): i) social credibility, which means people are more likely to recognize a source as believable if others recognize the source is believable, especially when there is not enough information to access the truthfulness of the source; ii) frequency heuristic, which means that people may obviously favour information they hear repeatedly, although it is fake news.

Based on these premise, the need for designing processes that automatically detect fake news calls for machine learning techniques that showed promising performances due to their ability to extract hidden information from data.

### Our system in a nutshell

Fake news detection problem can be formalized as a classification task thus requiring features extraction and model construction. The detection phase is a crucial task as it is devoted to guarantee users to receive authentic information. We will focus on finding clues from news contents, adding multimedia analysis and making some considerations about the dynamic component related to the diffusion of the news through social media and the users who spread this news trying to understand if there exists some common patterns. Our goal is to provide a benchmark of some existing approaches defined so far when fake news is intentionally written to mislead users by mimicking true news. More in detail, traditional approaches are based on verification by human editors and expert journalists but do not scale to the volume of news content that is generated in online social networks. As a matter of fact, the huge amount of data to be analyzed calls for the development of new computational techniques. It is worth noticing that, such computational techniques, even if the news is detected as fake, require some sort of expert verification before being blocked. In our framework, we perform an accurate pre-processing of news data and then we apply three different approaches. The first approach is based on classical classification approaches such as logistic regression that resulted the most effective in our framework as we implemented several approaches and we compared them as will be better explained in next sections. We also implemented different deep learning approaches that leverage neural network features for fake news detection. Finally, for the sake of completeness we implemented some multimedia approaches in order to take into account misleading images.

### Plan of the paper

The paper is organized as in the following. Section 2 outlines the state of the art of techniques for fake news detection, showing the most diffused machine/deep learning methodologies. Section 3 details the proposed framework for supporting our benchmark study. Section 4 describes the experimental setup, while Section 5 discusses the obtained results in terms of accuracy and efficiency. Eventually, we discuss the findings of our analysis in Section 6 while Section 7 reports some conclusions and future directions for our research.

## Related work

The fake news meaning has evolved over the time assuming nowadays the sense of any article or message propagated through media platforms carrying behind it false or misleading information (Sharma et al., 2019). Some well known examples of fake news across history are mentioned below:

- During the second and third centuries AD, false rumours were spread about Christians claiming that they engaged in ritual cannibalism and incest;^1^
- In 1835 The New York Sun published articles about a real-life astronomer and a fake colleague who, according to the hoax, had observed bizarre life on the moon;^2^
- More recently we can cite some news like, Paul Horner, was behind the widespread hoax that he was the graffiti artist Banksy and had been arrested; a man has been honored for stopping a robbery in a diner by quoting Pulp Fiction; and finally the great impact of fake news on the 2016 U.S. presidential election, according to CBS News.^3^

Thus, fake news deceive people by creating a false impression or conclusion (Lazer et al., 2018) whose detection is made difficult by the use of heterogeneous topics and different linguistic styles for their production (Shu et al., 2017). Rubin et al. (2015) organized the fake news into three categories: *serious fabrications*, being prototypical form of fake news that often become viral through social media, *large scale hoaxes*, representing false information disguised as proper news, and *humorous fakes*, having the aim to amuse readers. These fake news are spread on the network by an increasing number of malicious users, named *Spammer* (Bindu et al., 2018), whose detection is a challenging task although different approaches have been proposed (see Dewang and Singh 2018 for more details).

According to Bondielli and Marcelloni (2019), it is possible to classify approaches for fake news detection on the basis of the exploited features into *content* and *user*-based techniques. The former has the aim to classify news according to their inherent content (mainly news text) (Castelo et al., 2019), whilst the latter aims to deal with dynamic propagation of fake news according to user-based, text-based, propagation-based and temporal-based features (Castillo et al., 2011; Ma et al., 2015).

The content-based approaches aim to classify news according to their inherent content (mainly news text). Several machine learning models have been then proposed for analyzing information content and performing the related classification. Nevertheless, it is frequent to observe a performance slump because classical classifiers are not able to generalize and to classify instances never seen before as, instead, it can happen for fake news.

The most effective content-based methods rely on the *N*-grams, i.e. sequences of *N* contiguous words within a text (e.g., unigrams, bigrams, trigrams etc.). The first interesting approach leveraging such kind of features has been proposed by Mihalcea and Strapparava (2009) for lie detection using Naïve Bayes and SVM classifiers in order to identify people’s lies about their belief. More recently, Gilda (2017) analyzed 11,000 articles from several sources applying term frequency-inverse document frequency (TF-IDF) of bi-grams within a probabilistic context free grammar (PCFG) for fake news detection. The evaluation has been performed using different classification methods as Support Vector Machines, Stochastic Gradient Descent, Gradient Boosting, Bounded Decision Trees, and Random Forests. A very useful work is that proposed by Khan et al. (2019), where they studied the performances of different content-based approaches on various datasets, evaluating also several features as well as lexical, sentiment and *N*-grams ones. In turn, Jain and Kasbe (2018) proposed a specified method based on Naive Bayes classifiers with the aim to predict if a given post on Facebook is real or fake. Finally, in Kotteti et al. (2018) the authors tried to handle the missing values problem in fake news datasets by using data imputation for both categorical, with the most frequent values in the columns, and numerical features, using the mean value of the related column. In addition, TF-IDF vectorization was applied in feature extraction to unveil main features to use as input for a Multi-Layer Perceptron (MLP) classifier.

In turn, user-based features are typically used for classifying users in genuine or fake (Hu et al., 2014) that could be used as measure of the reliability of the shared information. Other features concerns information about social circles and activities made in Online Social Media, as well as number of posts, following/follower or their ratio (Zubiaga et al., 2016), or account’s age and/or linking to external resources (Wu et al., 2015; Zubiaga et al., 2016). Nevertheless, information about user’s activities on Online Social Networks cannot typically be gathered due to privacy constraints. According to Ma et al. (2017) different studies rely on network-oriented features for analyzing diffusion patterns (Ma et al., 2015; Hamidian and Diab, 2019) and modeling the temporal characteristics of propagation (Kwon et al., 2013).

Finally, some approaches (Vosoughi et al., 2017; Wang & Terano, 2015; Wu et al., 2015; Ma et al., 2015) have been proposed combining content and user based features for fake news detection. As an example, Castillo et al. (2011) proposed a machine learning approach based on decision tree model for classifying news as fake combining three different types of features: user-based (e.g. registration age and number of followers), text-based (e.g. the proportion of tweets that have a mention ‘@’), and propagation based (e.g. the depth of the re-tweet tree). The *FANG* framework has been proposed by Nguyen et al. (2020) for fake news detection by using social context through graph representation. Furthermore, Wang et al. (2020) designed a weakly-supervised fake news detection framework by combining news’ content and users’ report, that has been used in a reinforcement learning strategy for improving the obtained results. A further analysis of fake news detection has been discussed in Shu et al. (2019), in which the authors investigate the explainable detection of fake news by developing a deep architecture for jointly capturing top-k-check-worthy sentences and user comments.

Concerning benchmarks, different studies (Gravanis et al., 2019; Reis et al., 2019; Silva et al., 2020) have been designed to compare proposed approaches for fake news detection but they focused on small datasets and/or analyzed only some machine learning approaches.

For the paper aims, we have decided to focus on content-based techniques based both on machine and deep learning algorithms because: i) it is present in the literature a more systematic and wide study of such methods, ii) the adopted (and most diffused) datasets available for benchmarking mainly contain textual features of fake news. In addition, a real-time detection first of all has to consider news text before any other kind of user-based analysis that needs to observe the news temporal spread over social networks. Furthermore, we analyze some multimedia approaches in order to take into account misleading images and how they can be exploited to improve the overall fake news detection performances.

Summarizing, our analysis is focused on the detection of fake news at early stage, that is when it is published on a social media. For this reason, we only analyze news’ content with the aim to identify fake news, as also made in Gravanis et al. (2019); Reis et al. (2019) and Silva et al. (2020), without considering temporal and user-based features, that are typically used for contextual fake news detection (Nguyen et al., 2020; Wang et al., 2020).

Finally, the novelties of the proposed benchmark concern:

- a different pre-processing strategy with respect to Gravanis et al. (2019), that is mainly based on Natural Language Processing (NLP) pipeline;
- the analysis of a large number of machine and deep learning models with respect to Gravanis et al. (2019); Reis et al. (2019) and Silva et al. (2020), that are only based on well-known neural networks models;
- a deep analysis of an unbalanced large dataset (*FakeNews*) as well as two other smaller ones, whilst (Gravanis et al., 2019; Reis et al., 2019; Silva et al., 2020) rely only on small dataset (composed by 10,000 samples at maximum);
- a further analysis of some multimedia approaches in order to improve the overall fake news detection performances, also considering misleading images.

## Fake news detection: an experimental test bed framework

**Fig. 1:**
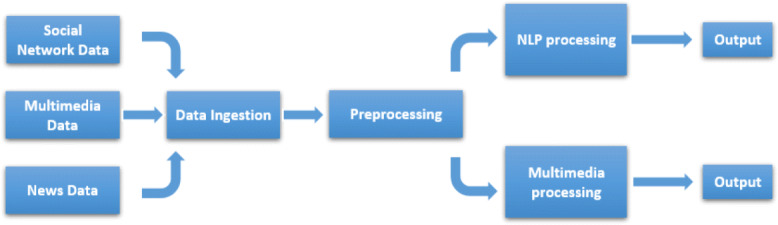
The overall process at a glance

We define a Fake News Detection framework for experimental purposes, based on news flow processing and data management, as depicted in Fig. 1. In particular, a preliminary pre-processing stage executes filtering and aggregation operation over the news content, and in addition filtered data are processed by two independent modules: the first one performs natural language processing over data, while the second one performs a multimedia analysis.

More in details:

- **Data Ingestion Module.** This module takes care of data collection tasks. Data can be highly heterogeneous as well as social network, multimedia and news data. We collect the news text and eventual related contents and images.
- **Pre-processing Module.** This component is devoted to the acquisition of the incoming data flow. It performs filtering, data aggregation, data cleaning and some enrichment operations.
- **NLP Processing Module.** It performs the crucial task of generating a binary classification of the news articles, i.e., whether they are fake or reliable news. It is split in two sub-modules. The *Machine Learning* module performs classification using an ad-hoc implemented algorithms after an extensive process of feature extraction and selection TF-IDF based (in order to reduce the number of extracted features). The *Deep Learning* module classifies data by different engines, after a tuning phase on the vocabulary. It also perform a binary transformation and eventual text padding in order to better analyze the input data.
- **Multimedia Processing Module.** This module is tailored for Fake Image Classification through Deep Learning algorithms, using ELA (Error Level Analysis) and CNN.

Due to space limitation, we discuss in the following only the details of the deep learning module bases on *Google B.E.R.T.* framework(Devlin et al., 2019), and the obtained results.

### Software architecture

**Fig. 2:**
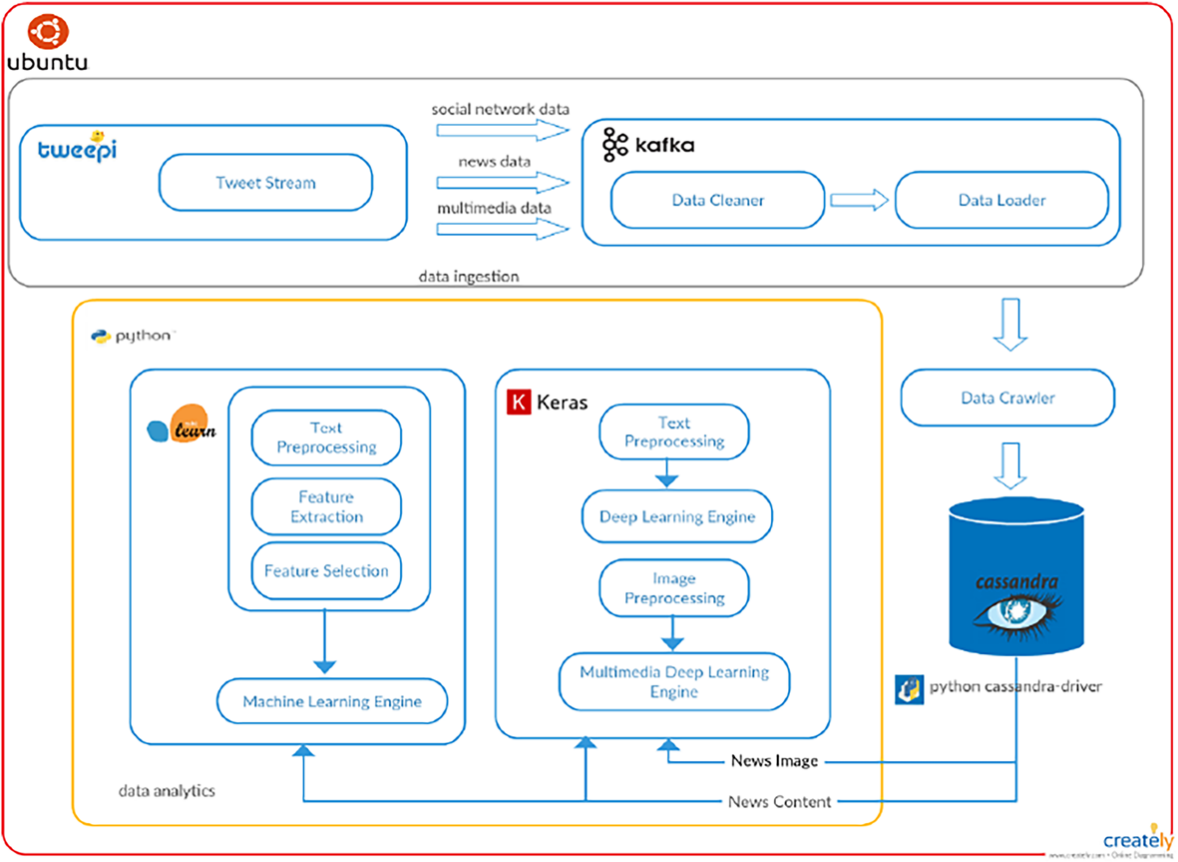
A fake news detection framework

The software implementation of the framework described above is shown in Fig. 2.

Herein: the data ingestion block is implemented by using several tools. As an example for Twitter data we leverage Tweepy,^4^ a Python library to access the Twitter API. All tweets are downloaded through this library. Filtering and aggregation is performed using Apache Kafka^5^ which is able to build real-time data pipelines and streaming apps. It is scalable, fault-tolerant and fast thus making our prototype well-suited for huge amount of data.

The data crawler uses the Newspaper Python library^6^ whose purpose is to extract and curate articles. The analytical data archive stores pre-processed data that are used for issuing queries by traditional analytical tools. We leverage Apache Cassandra^7^ as datastore because it provides high scalability, high availability, fast writing and fault-tolerance on commodity hardware or cloud infrastructure. The data analytics block retrieves news contents and news images from Cassandra DB that are pre-processed by the Machine Learning module using Scikit Learn library^8^ and by Deep Learning module using Keras library.^9^ Image content is processed by the Multimedia Deep Learning module using again Keras library. In the following we will briefly describe how the overall process is executed. Requests to the Cassandra DB are made through remote access. Each column in Cassandra refers to a specific topic and contains all news belonging to that topic. Among all news, those having a valid *external link* value are selected. In this way, the news content can be easily crawled. As the link for each news is obtained, a check is performed in order to verify the current state of the website. If the website is still running, we perform the article scraping. The algorithm works by downloading and parsing the news article, then, for each article, title, text, authors, top image link, news link data are extracted and saved as a JSON file in Cassandra DB.

Finally, three independent analysis are then performed by three ad-hoc Python modules we implemented. The first two perform text classification, and the last one images classification. Concerning the text analysis, the problem being solved is a binary classification one where class 0 refers to reliable news and class 1 refers to fake ones.

### The deep learning module in detail

The Deep Learning Module computes a binary classification on a text datasets of news that will be labelled as 0 if a news is marked as Real, and as 1 if it is marked as Fake. The Deep Learning Module classifies news content also exploiting a new language model called *B.E.R.T.* (Bidirectional Encoder Representations from Transformers) developed and released by Google. Prior to describing the algorithm features in detail, we briefly describe the auxiliary tools being used, while in Section 4 we describe the experimental evaluation that leads to our choice on B.E.R.T..

#### Colaboratory

Colab^10^ is intended for machine learning education and research, it requires no setup and runs entirely on the cloud. By using Colab it’s possible to write and execute code, save and share analytics and it provides access to expensive and powerful computing resources for free by a web interface.

More in detail, Colab’s hardware is powered by: Intel(R) Xeon(R) CPU @ 2.00GHz, nVidia T4 16 GB GDDR6 @ 300 GB/sec, 15GB RAM and 350GB storage. This setting is able to speed-up the learning task execution up to 35X and 16X faster in deep learning training compared to a CPU-only server.

#### Tensor flow

It is devoted to train and run neural networks for image recognition, word embeddings, recurrent neural networks, and natural language processing. It is a cross-platform tool and runs on CPUs, GPUs, even on mobile and embedded platforms. TensorFlow(Abadi et al., 2016) uses dataflow graphs to represent the computation flow, i.e., these structures describe the data flow through the processing nodes. Each node in the graph represents a mathematical operation, and each connection between nodes is a multidimensional data array called tensor.

The TensorFlow Distributed Execution Engine abstracts from the supported devices and provides a high performance core implemented in C++ for the TensorFlow platform. On top there are Python and C++ front ends. The Layers API provides a simple interface for most of the layers used in deep learning models. Finally, higher-level APIs, including Keras, makes training and evaluating distributed models easier.

#### Keras

It is a high-level neural network API,^11^ implemented in Python and capable of running on top of TensorFlow. It allows for easy and fast prototyping through: 1) User Friendliness as it offers consistent and simple APIs that minimizes the number of user actions required for common use cases; 2) Modularity as neural layers, cost functions, optimizers, initialization schemes, activation functions and regularization schemes are all standalone modules that can be combined to create new models; 3) Extensibility as new modules are simple to add as new classes and functions.

#### Google B.E.R.T.

This tool has been developed in order to allow an easier implementation of two crucial tasks for Natural Language Processing (NLP): Transfer Learning through unsupervised pre-training and Transformer architecture. The idea behind Transfer Learning is to train a model in a given domain on a large text corpus, and then leverage the gathered knowledge to improve the model’s performance in a different domain. In this respect, B.E.R.T.^12^ has been pre-trained on Wikipedia and BooksCorpus. On the opposite side, the Transformer architecture processes all elements simultaneously by linking individual elements through a process known as attention. This mechanism allows a deep parallelization and guarantee higher accuracy across a wide range of tasks.^13^ B.E.R.T. outperforms previous proposed approaches as it is the first unsupervised, fully bidirectional system for NLP pre-training. Pre-trained representations can be:

- *context-free*: this representation generates a single word embedding representation for each word in the vocabulary, so bank would have the same representation in bank deposit and river bank;
- *contextual*: in this case a representation of each word that is based on the other words in the sentence is generated.

B.E.R.T. was built based on recent work in pre-training contextual representations, such as ELMo or ULMfit but these models are mainly unidirectional. This means that each word is only contextualized using the words to its left (or right). For example, in the sentence “I made a bank deposit” the unidirectional representation of “bank” is based either on “I made a” piece of text or “deposit” piece of text. B.E.R.T. represents the term “bank” using both its left and right context “I made a $\dots $ deposit”. This feature allows the model to learn the context of a word based on the whole sentence (left and right of the word).

B.E.R.T.’s model architecture is based on a multi-layer bidirectional Transformer Encoder, an attention mechanism that learns contextual relations between words (or sub-words) in a text. Transformer includes two different mechanisms — an encoder that reads the input text and a decoder that produces a prediction for the task. Since B.E.R.T.’s goal is to generate a language model, only the encoder mechanism has to be properly manipulated.

**Fig. 3:**
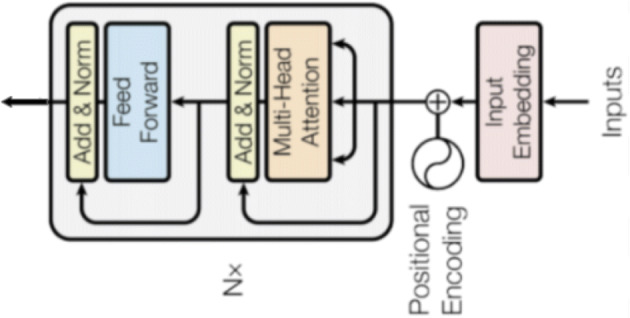
The Econder input

The Encoder’s input embedding depicted in Fig. 3 and it is composed by: i) token embeddings: it represents the word vector. The first word is the CLS token that is used as a delimiter. It can be used for classification tasks, on the contrary for non-classification tasks, the CLS token can be ignored; ii) segmentation embeddings: it is used to distinguish between two sentences as pre-training can be seen a classification task with two sentences as input; iii) position embeddings: it encodes word ordering.

The data flow through the Encoder Architecture is described in what follows: 1) The model represents each token as a vector of *emb*_*dim* size (dimension of the token embeddings). By assigning one embedding vector for each of the input tokens, we obtain a matrix whose dimensions are *input*_*length* and *emb*_*dim* for each input sequence; 2) It then adds positional information (positional encoding). The approach chosen is to add values between [-1,1] using predetermined (non-learned) sinusoidal functions to the token embeddings. Words will be represented slightly differently depending on their position (even for same word). This step builds again a matrix having dimensions *input*_*length* and *emb*_*dim*. 3) Data are elaborated by N encoder blocks. Each encoder block is Multi-Head Attention layer that computes *h* different attention values by different weight matrices and then concatenates the results. This step allows the model leverage different representation sub-spaces for different word positions and the use of different filters to create different features maps in a single layer.

Its purpose is to find relationships between the input representations and encode them in output. After this step, we obtain a vector of hidden size (768 in B.E.R.T. Base and 1024 in B.E.R.T. Large). This vector is used as input on a single-layer neural network classifier to obtain the final output.

After pre-elaboration B.E.R.T. works in two steps: *pre-training*, i.e., the model is trained on unlabelled data over different tasks and *fine-tuning*, i.e., the B.E.R.T. model initialized with the pre-trained parameters is fine-tuned using labelled data from the downstream tasks. Each downstream task has separate fine-tuned models, even though they are initialized with the same pre-trained parameters.

The B.E.R.T. pre-training phase consists of two unsupervised predictive tasks: 1) *Masked Language Model*: 15% of the words in each sequence are replaced with a *MASK* token. The model then attempts to predict the original value of the masked words, based on the context provided by the other, non-masked, words in the sequence. For example, if the input of the neural network is “I came to [MASK] and bought [MASK]”, it should show the words “store” and “milk” in output; 2) *Next Sentence Prediction*: the model receives pairs of sentences as input and learns to predict if the second sentence in the pair is the subsequent sentence of the first one in the original document. During training, 50% of the inputs are pairs having the second sentence as correct subsequent sentence in the original document, while in the 50% are random sentences chosen from the corpus. The assumption is that the random sentence will be unrelated from the first sentence. For example, given two sentences “I went to the store.” and “And bought milk there.”, the neural network should answer that this is a relaible consequent. On the contrary, if the second phrase is “Cruc’s sky Pluto” then it should answer that this sentence is not related to the first. During the fine-tuning phase, B.E.R.T. can be used for a wide variety of language processing task by implementing an additional layer for the model. As and example, it is possible to perform classification tasks such as sentiment analysis by adding a classification layer on top of the Transformer output for the CLS token. Moreover, for Question Answering (QA) tasks (e.g. SQuAD v1.1), the model is queried with a question regarding a text sequence and is required to mark the correct answer in the sequence. Using B.E.R.T., a QA model can be trained by learning two extra vectors that mark the beginning and the end of the answer. Finally, Named Entity Recognition (NER) task aims to mark the entity types (e.g., Person, Organization, Date, and so on) that appear in the text. Using B.E.R.T., a NER model can be trained by giving the output vector of each token as input of a classification layer that predicts the NER label.

**Fig. 4:**
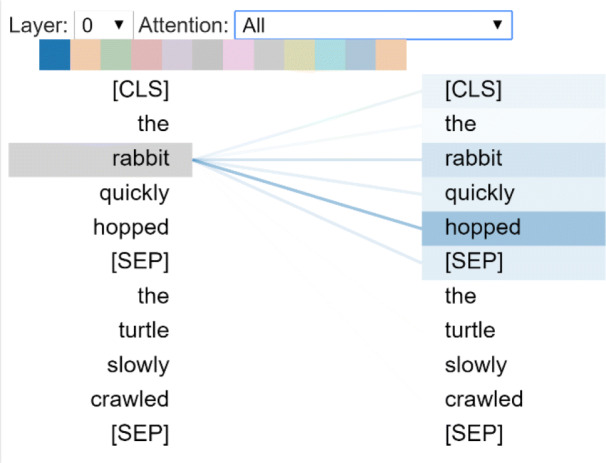
B.E.R.T. attention-head view

Furthermore, B.E.R.T. is able to build composite data representations to understand language features by Attention mechanism. This task is performed by BertViz, an interactive tool that visualizes attention pattern in B.E.R.T. from multiple perspectives, i.e., Attention-Head View and Multi-Head Attention View. In Attention-Head View the visualization shows the attention induced by a sample input text. This view visualizes attention as lines connecting the word being updated (left) with the word being attended to (right) as shown in Fig. 4.

Colours encode the attention weight: weights close to one are represented as darker lines, while weights close to zero appear as almost invisible.

The example depicted in Fig. 4 is based on two sentences: “the rabbit quickly hopped” and “the turtle slowly crawled”. The *SEP* symbols are special tokens used as sentence delimiters, and *CLS* has the meaning described above. The visualization shows that attention is highest between words that do not cross a sentence boundary; the model seems to understand that it should relate words to other words in the same sentence in order to understand their context.

Multi-Head Attention in B.E.R.T. learns multiple attention mechanisms, called heads, which operate in parallel enabling the model to capture a broader range of relationships between words. As the attention heads do not share parameters, each head learns a unique attention pattern.

#### The machine learning module

As mentioned above the goal of the Machine Learning Module is to produce a binary classification on a text dataset. Thus, a news article will be labelled as 0 if it is recognised as *Real*, and as 1 if it is recognised as *Fake*. We devise a supervised approach since the dataset we worked on is fully labelled. The Machine Learning implementation has been chosen by comparing most of the available classifiers provided by the Scikit- Learn library.

It has been developed using a Python 3 kernel in Jupyter, that is a web-based interactive development environment for code, and data. It is flexible, extensible, modular and configurable to support a wide range of workflows in data science, scientific computing, and machine learning. We choose Python as it is interactive, interpreted, modular, dynamic, object- oriented, portable and extensible thus offering an high flexibility for our purposes.

More in detail, the following libraries have been used: i) Scikit- Learn: a simple and efficient tool for data mining and data analysis; ii) Spacy: an open-source software library for advanced Natural Language Processing, iii) Numpy: a library for Python programming language that offer support for large, multi- dimensional arrays and matrices, along with a large collection of high level mathematical functions to operate on these arrays, iv) Pandas: an open source, BSD-licensed library providing high-performance, easy-to-use data structures and data analysis tools for the Python programming and v) Matplotlib: a plotting library for the Python programming language and its numerical mathematics extension NumPy.

### The multimedia module

This module performs a Fake Image Classification by using a Deep Learning model based on Error Level Analysis (ELA) and Convolutional Neural Networks (CNN), whose aim is to find if an image has been manipulated or not Thus, an image related to a news article, will first be submitted to an ELA and then will be labelled as 0 if it is recognised as Real, i.e., it has been not manipulated, and as 1 if it is recognised as Fake, i.e., it has been manipulated. The Multimedia Deep Learning Module has been developed using a Python 3 kernel in a Jupyter. For the implementation, the above describes libraries have been used: Keras, Scikit and Numpy. Moreover, we leveraged the modules described below.

#### Pandas

It is an open source, BSD-licensed library providing high-performance, easy-to-use data structures and data analysis tools for the Python programming.

#### Pillow (PIL)

It is the Python Imaging Library, a free library for the Python programming language that adds support for accessing and manipulating several different image file formats.

#### Numpy

It is a library for Python programming language, tailored for large multi-dimensional arrays and matrices manipulation by a huge collection of high-level mathematical functions.

#### Matplotlib

It is a plotting library for the Python programming language and its numerical mathematics extension NumPy.

#### Parameter setting

CNN are complex networks that require many hyper parameters to be set as their values heavily affect the quality of the obtained results. As a matter of fact, the tuning phase requires many tests to be conducted in order to find optimal parameter assignments. In our framework, we manipulated the hyper parameters reported below:

- Architecture-Type and number of hidden layers: the number of hidden layers defines the depth of the network. The depth of the proposed layers has been consistently increased and in general performs better than a shallow network;
- Optimizers: the selected optimizers for investigation are Momentum, RMSProp and Adam. After a deep experimental evaluation we choose Adam;
- Activation Function: the activation function used is ReLU. For binary-classification Sigmoid and Softmax can be used for the last layer. In our framework we choose Sigmoid;
- Dropout Regularization: a regularization technique which avoid overfitting during the training;
- Convolution Layer: there are many parameters that can be changed, however, it is the number of kernels applied to each layer, the height and width of each convolutional kernel and padding;
- Dimensions of pooling matrix: the most commonly used size for pooling is 2x2, i.e., images are half down sampled. A larger pooling matrix size would increase the down sampling rate;
- Number of Epochs: defines the number times that the learning algorithm will work on the entire training dataset. We set this value to 10;
- Batch size: defines the number of samples used, before updating the internal model parameters. Possible values are 16, 32, 64. We found in our experiments that the optimal value is 32.

## Experimental setup

This section aims to describe the experimental setup for the adopted benchmark system.

### Dataset

We analyzed different Fake News datasets, publicly available, that differ in quantity, type of news and sentence length. In particular, we focused our attention on: *Liar*, *FakeNewsNet* and PHEME Datasets (Wang, 2017; Shu et al., 2018) that are described in details in what follows.

#### Liar dataset

This dataset includes 12.8K human labelled short statements from fact-checking website Politifact.com. Each statement is evaluated by a Politifact.com editor for its truthfulness. The dataset has six fine-grained labels: pants-fire, false, barely-true, half-true, mostly-true, and true. The distribution of labels is relatively well- balanced. For our purposes the six fine-grained labels of the dataset have been collapsed in a binary classification, i.e., label 1 for fake news and label 0 for reliable ones. This choice has been made for comparison purposes due to binary Fake News Dataset feature. The dataset is partitioned into three files: 1) Training Set: 5770 real news and 4497 fake news; 2) Test Set: 1382 real news and 1169 fake news; 3) Validation Set: 1382 real news and 1169 fake news.

**Fig. 5:**
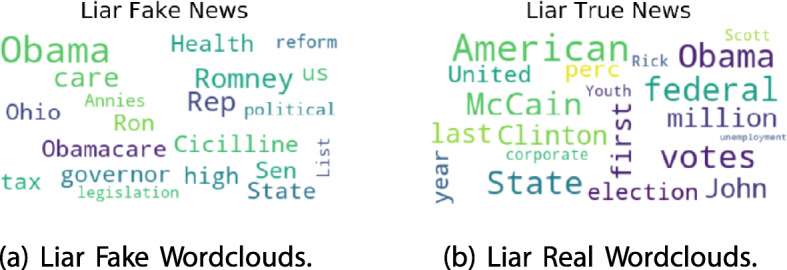
LIAR Fake (**a**) and Real (**b**) Wordclouds

The three subsets are well balanced so there is no need to perform oversampling or undersampling. The corresponding Wordclouds for fake news is reported in Fig. 5a. It is easy to see that news are mainly related to United States. Fake news topics are collected about Obama, Obamacare, Cicilline, Romney.

On the other side real news topics depicted in Fig. 5b refer to McCain, elections and Obama.

The processed dataset has been uploaded in Google Drive and, then, loaded in Colab’s Jupyter as a Pandas Dataframe. It has been added a new column with the number of words for each article row. Using the command *df*.*describe*() on this column it is possible to print the following statistical information: count 15389.000000, mean 17.962311, std 8.569879, min 1.000000, 25% 12.000000, 50% 17.000000, 75% 22.000000, max 66.000000. These statistics show that there are articles with only one word in the dataset, so it has been decided to remove all rows with less than 10 words as they are considered poorly informative. The resulting dataset contains 1657 less rows than the original one. The updated statistics are reported in what follows: count 13732.000000, mean 19.228663, std 8.192268, min 10.000000, 25% 14.000000, 50% 18.000000, 75% 23.000000, max 66.000000. Finally, the average number of words per article is 19.

#### FakeNewsNet

This dataset has been built by gathering information from two fact-checking websites to obtain news contents for fake news and real news such as PolitiFact and GossipCop. In PolitiFact, journalists and domain experts review the political news and provide fact-checking evaluation results to claim news articles as fake or real. Instead, in GossipCop, entertainment stories, from various media outlets, are evaluated by a rating score on the scale of 0 to 10 as the degree from fake to real. The dataset contains about 900 political news and 20k gossip news and has only two labels: true and false [14].

This dataset is publicly available using the functions provided by the FakeNewsNet team and the Twitter API. As mentioned above, FakeNewsNet can be split in two subsets: GossipCop and Politifact.com. We decided to analyse only political news as they produce worse consequences in real world than gossip ones. The dataset is well balanced and contains 434 real news and 367 fake news. Most of the news regards the US as it has been evaluated also in LIAR. Fake news topics concern Obama, police, Clinton and Trump while real news topics refer to Trump, Republicans and Obama. Such as the LIAR dataset, it has been added a new column and used the command *df*.*describe*() to print out the following statistical information: count 801, mean 1459.217228, std 3141.157565, min 3, 25% 114, 50% 351, 75% 893, max 17377.

The average number of words per articles in Politifact dataset is 1459, which is far longer than the average sentence length in Liar Dataset that is 19 words per articles. Such a statistics suggested us to compare the model performances on datasets with such different features.

Moreover, among the available columns we can access *main* − *img* that containis the URL of to the main image in the article. The latter feature allows us to use this dataset also for multimedia analysis. After a preliminary initial check relating to the validity of the URL provided by the dataset, the image file has been downloaded and stored for multimedia analysis.

#### PHEME Dataset

Among the available labelled datasets containing both real and fake news with related image, we used PHEME Dataset to train the classifier as it contains several news categories from politics to general news. The original dataset is partitioned into nine folders containing breaking news events. It is structured as follows: each event has a directory, with two subfolders, rumours and non-rumours. These two folders contain additional folders named with a news ID and each of these contains two different file: *annotation.json*, which contains information about veracity of the rumour and *structure.json*, which contains information about structure of the conversation. In order to obtain a single dataset, all the fake and real news articles regarding all 9 events, have been imported by Jupyter, loaded as Pandas Dataframe and stored in a CSV file. The resulting dataset contains 15k news articles with the main features linked to it along with the URLs of the corresponding files. Each of them has a label which is 0 if the statement and, consequently the image, is *Real*, or *Not Manipulated*, and 1 if the statement, and the image, is *Fake*, or *Manipulated*.

Finally, the dataset is partitioned as follows:

1. Training Set: 1779 real image and 2143 fake image;
2. Validation Set]: 771 real image and 895 fake image;
3. Test Set: 771 real image and 895 fake image.

Table 1 summarize the description of datasets in terms of number of samples chosen for training, test and validation steps.

**Table 1:** Dataset characterization

Dataset	Training		Test		Validation	
	Real	False	Real	False	Real	False
Liar	5,770	4,497	691	584	691	584
FakeNews	249,387	112,208	83,129	37,403	83,129	37,403
PHEME	1779	2143	771	895	771	895

### Pre-elaboration steps

The above mentioned datasets are available in CSV format and are composed of two columns: text and label. The news text need to be pre-processed for our analysis. In this respect, an ad-hoc Python function has been developed for unnecessary IP and URL addresses removal, HTML tags checking and words spell-check. Due to neural features, we decide to maintain some stop words in order to allow a proper context analysis. Thus, to ameliorate the noise problem, we created a custom list of stop words. We leverage Keras Tokenizer for preparing text documents for subsequent deep learning steps. More in detail, we create a vocabulary index based on word frequency, e.g., given the sentence *The cat sat on the mat* we create the following dictionary *word*_*index*[the] = 1, *word*_*index*[cat] = 2 so each word gets a unique integer value; the 0 value is reserved for padding. Lower integer means more frequent word. After this encoding step, we obtain for each text a sequence of integers. As B.E.R.T. needs a more elaborated input than other neural networks we need to produce a *tsv* file, with four columns, and no header. The columns to be added to dataset are: 1) *guid*, i.e., a row ID; 2)*label*, i.e., the label for the row (it should be an int); 3) *alpha*, a dummy column containing the same letter for all rows, it is not used for classification but it is needed for proper running of the algorithm and 4) *text*, i.e., the news content. The data needs to be converted in InputFeature object to be compatible with Transformer Architecture. The conversion process includes tokenization and converting all sentences to a given sequence length (truncating longer sequences, and padding shorter sequences). Tokenization is performed using WordPiece tokenization, where the vocabulary is initialized with all the individual characters in the language, and then the most frequent/likely combinations of the existing words in the vocabulary are iteratively added. Words that does not occur in the vocabulary are broken down into sub-words in order to search for possible matches in the collection.

#### Choosing and tuning the most suitable machine learning model

In order to choose the most suitable classification method, we performed an extensive tuning phase by comparing several algorithms. The performances have been evaluated on several test sets by comparing several accuracy measure like Accuracy, Precision, Recall, F1 measure, Area Under Curve (AUC) (Flach and Kull, 2015) (reported in Table 2) and execution times (reported in Table 5). Tables 3 and 4 summarize the obtained results on the two different datasets.

**Table 2:** Classifier effectiveness comparison

Classifier	Accuracy	Precision	Recall	F1	TP	FP	TN	FN	AUC
SGD	0.06	0.03	0.738	0.678	1,020	605	564	362	0.610
Naive Bayes	0.02	0.004	0.871	0.704	1,205	833	336	177	0.580
Linear SVC	0.27	0.002	0.662	0.642	915	553	616	467	0.595
Random Forest	3.57	0.06	0.790	0.674	1,093	765	404	289	0.568
Logistic Regression	0.22	0.002	0.758	0.684	1,034	603	566	348	0.616
Nearest Neighbor	0.019	4.17	0.646	0.616	894	625	544	488	0.556
Decision Tree	9.18	0.009	0.616	0.604	852	585	584	530	0.558
Gradient Boost	22.7	0.02	0.860	0.701	1,189	818	351	193	0.580
Perceptron	0.08	0.05	0.749	0.638	1,088	770	399	294	0.571
Passive Aggressive	0.09	0.04	0.768	0.702	935	533	586	497	0.591

**Table 3:** Results on LIAR for Machine Learning with basic parameters

Classifier	ACC	PRE	REC	F1	AUC	Training Time [s]	Test Time [s]
SGD	62.0%	62.7%	73.8%	67.8%	61.0%	0.06	0.03
Naïve Bayes	60.4%	59.1%	87.1%	70.4%	58.0%	0.02	0.004
Linear SVC	60.0%	62.3%	66.2%	64.2%	59.5%	0.27	0.002
Random Forest	58.6%	58.8%	79.0%	67.4%	56.8%	3.57	0.06
Logistic Regression	62.7%	63.1%	75.8%	68.4%	61.6%	0.22	0.002
Nearest Neighbor	56.3%	58.8%	64.6%	61.6%	55.6%	0.019	4.17
Decision Tree	56.2%	59.2%	61.6%	60.4%	55.8%	9.18	0.009
Gradient Boost	60.3%	59.2%	86.0%	70.1%	58.0%	22.7	0.02
Perceptron	57.8%	57.2%	74.9%	63.8%	57.1%	0.08	0.05
Passive Aggressive	59.3%	59.9%	76.8%	70.2%	59.1%	0.09	0.04

**Table 4:** Results on Polifact for Machine Learning with basic parameters

Classifier	ACC	PRE	REC	F1	AUC	Training Time [s]	Test Time [s]
SGD	58.3%	61.4%	72.2%	66.4%	59.9%	0.08	0.04
Naïve Bayes	57.9%	57.6%	85.6%	68.9%	56.7%	0.03	0.006
Linear SVC	55.6%	61.0%	65.0%	62.9%	58.3%	0.31	0.004
Random Forest	55.1%	57.4%	77.7%	66.0%	55.3%	3.59	0.08
Logistic Regression	59.6%	62.3%	74.9%	68.0%	60.2%	0.25	0.005
Nearest Neighbor	53.3%	57.5%	63.3%	60.3%	54.5%	0.021	4.20
Decision Tree	54.1%	57.8%	60.2%	60.0%	54.5%	9.20	0.012
Gradient Boost	57.7%	57.7%	84.7%	68.6%	56.8%	22.9	0.05
Perceptron	53.9%	55.8%	73.2%	63.3%	55.7%	0.10	0.06
Passive Aggressive	55.2%	58.5%	75.5%	65.9%	58.0%	0.11	0.05

As it is easy to see, the best model in terms of accuracy turns out to be Logistic Regression, so we decided to perform a parameter optimization for this algorithm as it exhibits the best results on each efficiency and effectiveness measure. It is worth noticing that, classifiers based on tree construction executes much slower because of the training step (Table 5).

**Table 5:** Execution times comparison

Classifier	Training Time	Test Time
SGD	0.06	0.03
Naive Bayes	0.02	0.004
Linear SVC	0.27	0.002
Random Forest	3.57	0.06
Logistic Regression	0.22	0.002
Nearest Neighbor	0.019	4.17
Decision Tree	9.18	0.009
Gradient Boost	22.7	0.02
Perceptron	0.08	0.05
Passive Aggressive	0.09	0.04

We briefly recall here, that logistic regression is a statistical model that leverages the logit function to model a binary dependent variable, i.e., a linear combination of the observed features:

$$ log \frac{p}{1-p} = \beta_{0} + \beta_{1} \dot x $$

Logistic Regression outputs the probabilities of a specific class that are then used for class predictions. The logistic function exhibits two interesting properties for our purposes: 1) it has a regular “s” shape; 2) Its output is bounded between 0 and 1.

Compared with other models, Logistic Regression offers the following advantages: 1) it is easily interpretable; 2) Model training and prediction steps are quite fast; 3) Only few parameters has to be tuned (the regularization parameter); 4) It outputs well-calibrated predicted probabilities.

In order to tune the algorithm we leveraged the functionalities offered by SciKit.

#### B.E.R.T. parameter tuning

In Table 6, we show the basic parameter assignment that is widely used for training.

**Table 6:** Basic parameter setting for deep learning networks

Parameter	Value
Number of Epoch	10
Batch size	32
Input Length	100
Validation Split	0.2

After a fine tuning of the B.E.R.T. parameters on Liar and FakeNews datasets, we found the best setting for running the experiments that are reported in Table 7.

**Table 7:** Model settings B.E.R.T. optimization

Parameter	Value
Model	GoogleBERT
Feature	Transformer Word Embeddings
Stop Words	Partially Removed
Batch size	32
Learning Rate	2*e*^− 5^
Max Sequence Length	160
Warm up	0.1
Tune Cell	0

#### Multimedia neural network parameter tuning

In Table 8 we report an excerpt of our setting steps on PHEME dataset (similar results have been obtained on the other datasets). We fixed the following parameter for CNN1: Number of Epoch = 10; Batch size = 32; Learning Rate = 0.001; Pooling matrix = 2x2; Dropout= 0.5; Input Shape = (128,128) and Activation Function= ReLU. We compared the performances on well-established evaluation measure like: Accuracy, Precision, Recall, F1 measure, Area Under Curve (AUC) (Flach & Kull, 2015) and the values reported in the obtained confusion matrices for each algorithm, i.e., True Positive (TP), False Positive (FP), True Negative (TN) and False Negative (FN).

**Table 8:** Parameter tuning for pheme dataset

CNN	Accuracy	Precision	Recall	F1	TP	FP	TN	FN	AUC
CNN #1	0.512	0.482	0.437	0.458	302	324	651	389	0.501
CNN #2	0.623	0.568	0.597	0.582	549	418	329	370	0.662
CNN #3	0.745	0.719	0.676	0.697	571	223	598	274	0.741
CNN #4	0.758	0.740	0.770	0.755	598	210	680	178	0.753

In order to try to improve the accuracy we changed for CNN2 the learning rate to 0.001 and the activation function as sigmoid. The results showed a 20% improvement in accuracy. The latter is due to the new optimizer value, which combines the heuristics of both Momentum and RMSProp, and the different function used for the last layer, which performs better in binary-classification. To further improve the results, we implemented CNN3 by adding two additional layers that caused a further accuracy increase. Finally, we used a (3x3) kernel sizes that results in a lower number of weights and higher number of layers that turns out to be a more computationally efficient choice. Hence, we can conclude that 3x3 convolution filters will be a better choice.

## Experimental results

### Machine learning approach evaluation

In this section we reported the results of machine learning, deep learning and multimedia tailored approaches implemented in our benchmark.

First, we performed a parameter optimization for the choosen Logistic Regression Algorithm, obtaining the results reported in Table 9.

**Table 9:** Results of optimized logistic regression on politifact and LIAR datasets

DATASET	ACC	PRE	REC	F1	AUC
LIAR	63.5%	62.9%	79.6%	70.2%	62.1%
POLIFACT	62.1%	61.7%	79.2%	69.4%	60.6%

**Fig. 6:**
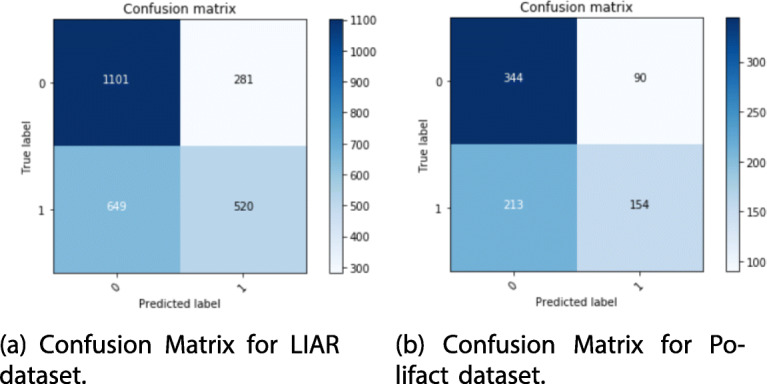
Confusion Matrix for LIAR (**a**) and Polifact (**b**) datasets

For the sake of completeness, we report in Fig. 6a and b the detailed confusion matrices obtained for LIAR and Polifact datasets.

We hypothesize that our results are quite better due to the fine feature selection task we performed, a better pre-processing step and the proper text transformation and loading.

**Fig. 7:**
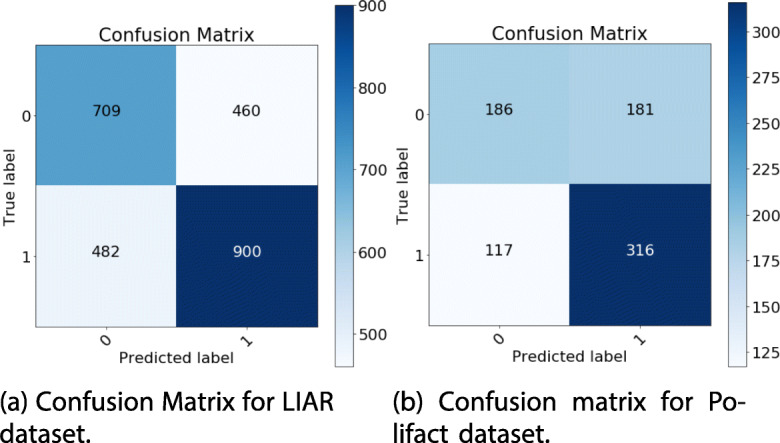
Confusion Matrix for LIAR (**a**) and Polifact (**b**) datasets

### Deep learning evaluation

As mentioned above, the deep neural networks evaluation has been initially performed by training the models using the basic parameters in Table 6. The performances evaluated on Liar and PolitiFact datasets are shown in Tables 10 and 11.

**Table 10:** Results on LIAR for deep learning with basic parameters

Neural Network	ACC	PRE	REC	F1	AUC	Training Time	Test Time
B.E.R.T.	61.9%	58.3%	59.6%	62.8%	61.7%	525.15s	8s
C-HAN	55.7%	51.4%	62.8%	56.5%	57.4%	600.64s	11.85s
BI-LSTM	58.6%	55.4%	49.5%	52.3%	60.7%	483.24s	7.48s
CNN	53.6%	48.9%	27.5%	35.2%	53.9%	11.98s	0.28s

**Table 11:** Results on PoliFact for deep learning with basic parameters

Neural Network	ACC	PRE	REC	F1	AUC	Test Time
B.E.R.T.	58.8%	56.5%	44.9%	62.8%	57.8%	8s
C-HAN	44.8%	44.3%	79.5%	56.9%	43.6%	11.85s
BI-LSTM	51.1%	46.6%	45.5%	46.0%	53.2%	7.48s
CNN	54.0%	49.8%	40.5%	44.7%	52.0%	0.28s

As easy to note, Google B.E.R.T. obtained the overall best results both on Liar Test and PolitiFact Tests. Nevertheless, as mentioned in previous section, we performed a parameter optimization of B.E.R.T. network (reported in Table 7) for analyzing the (eventual) performance improvement. More in details, Table 12 shows that the performances increase in terms of accuracy, precision and AUC

**Table 12:** Results of Deep Learning B.E.R.T. on Polifact and LIAR datasets

DATASET	ACC	PRE	REC	F1	AUC
LIAR	63.0%	59.5%	60.6%	62.8%	62.8%
POLIFACT	62.7%	61.3%	50.6%	62.8%	61.8%

We hypothesize that our results are quite better due to the fine hyper parameter tuning we performed, a better pre-processing step and the proper transformation we leverage. For the sake of completeness, we report in Fig. 7a and b the detailed confusion matrices obtained for LIAR and Polifact datasets.

### Multimedia approach evaluation

**Fig. 8:**
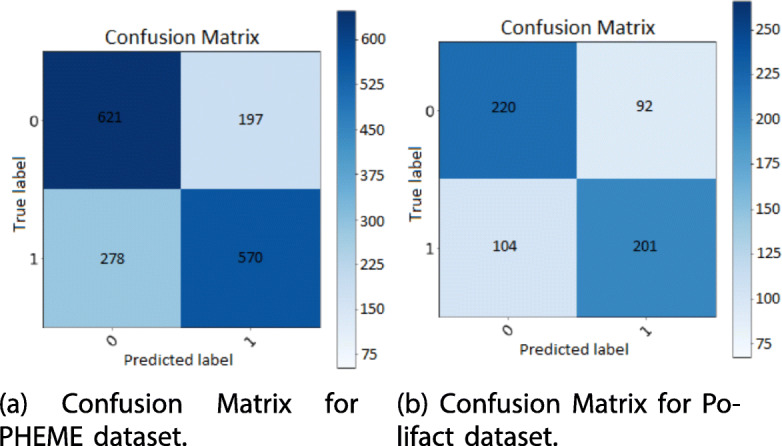
Confusion Matrix for PHEME (**a**) and Polifact (**b**) datasets

After the tuning phase described in previous section, we performed our experiments on PHEME dataset by leveraging CNN4 whose results are reported in Fig. 8a.

We hypothesize again, that our results are quite better due to a fine hyper parameter tuning we performed, a better pre-processing step and the proper transformation. For the sake of completeness, we report in Table 13 and Fig. 8b the accuracy measures and confusion matrix obtained by CNN4 on Polifact datasets.

**Table 13:** Evaluation measures for polifact dataset

CNN	Accuracy	Precision	Recall	F1	TP	FP	TN	FN	AUC
CNN #4	0.765	0.679	0.705	0.692	220	104	201	92	0.751

## Discussions

In this paper we designed a benchmark framework in order to analyze and discuss the most widely used and promising machine/deeplearning techniques for fake news detection, also combining different features. More in details, our analysis is focused on the detection of fake news at early stage, that is when it is published on a social media. For this reason, we only analyze news’ content with the aim to identify fake news, as also made in Gravanis et al. (2019); Reis et al. (2019) and Silva et al. (2020), without considering temporal and user-based features, that are typically used for contextual fake news detection (Nguyen et al., 2020; Wang et al., 2020).

We performed our analysis on three dataset (an unbalanced large dataset (*FakeNews*) as well as two other smaller ones (*PHEME* and *LIAR*)). Firstly, a comparison among different machine learning models (i.e. Random Forest, Decision Tree, SVC and Logistic Regression) is performed, which highlighted Logistic Regression as the best model in terms of efficiency and efficacy measures. Compared with other models, Logistic Regression offers different advantages such as interpretability, fast execution time and few parameters to be tuned. Successively, deep learning models (i.e. Convolutional Neural Networks and BERT) are compared. As easy to note in Tables 10 and 11, Google B.E.R.T obtained the overall best results because it performs word-level embedding on the basis of their context although it is complex to train. Finally, a multimodal strategy has been further investigated by combining content and multimedia analysis in order to perform a fake image classification. As can be seen in Table 13, this approach achieves the best results in terms of accuracy, precision, recall and F1 leveraging multimedia data.

## Conclusion

Fake news is a challenging task even though several techniques have been developed over time to mitigate their negative effects.

In this work, a benchmark analysis of fake news detection using classical Machine Learning and Deep Learning approaches (both for texts and images) has been discussed. As shown in Section 5 traditional machine learning classifiers have still advantages and some drawbacks. First, these methods are very fast both during training and test steps for supporting real time analysis. On the other hand, these methods are not still able to detect words semantic meaning and context of the word picked up from a sentence obtaining low accuracy values.

In turn, deep learning classifiers can automatically extract textual features and analyze semantic meaning of the words based on sentence context and images. Nevertheless, these neural networks are still slower in training time than traditional machine learning thus requiring a very powerful hardware to be able to work properly at social network scale. Thus, although hardware limitations, results obtained are very promising and there is still further improvement to achieve.

We are planning to consider in the future more different data sets composed by a growing number of samples and various topics; eventually, we are also planning to analyze various architectural design for Real-time fake news detection, and related methods.

## Data Availability

Three different dataset have been investigated in the proposed analysis: *Liar*,^14^*FakeNews*^15^ and PHEME^16^

## Footnotes

1 https://en.wikipedia.org/wiki/Fakenews

2 http://www.snelgraphix.net/the-snelgraphix-designing-minds-blog/tag/google+I%E2%80%99m+feeling+stellar

3 https://www.businessinsider.com/banksy-arrest-hoax-2013-2

4 https://www.tweepy.org/

5 https://kafka.apache.org/

6 https://newspaper.readthedocs.io/en/latest/

7 http://cassandra.apache.org/

8 https://scikit-learn.org/stable/

9 https://keras.io/

10 https://research.google.com/colaboratory

11 https://keras.io

12 https://github.com/google-research/bert

13 https://towardsdatascience.com/deconstructing-bert-part-2-visualizing-the-inner-workings-of-attention-60a16d86b5c1https://towardsdatascience.com/deconstructing-bert-part-2-visualizing-the-inner-workings-of-attention-60a16d86b5c1
